# Appel pour une allocation intelligente dans le financement du système de santé au Cameroun

**DOI:** 10.4314/pamj.v9i1.71179

**Published:** 2011-05-23

**Authors:** Isidore Sieleunou

**Affiliations:** 1 Ministère de la Santé Publique, Yaoundé, Cameroun

**Keywords:** Financement, budget, système de santé, économie, Cameroon

## Abstract

Le Cameroun utilise un système de budgétisation historique et une approche «top-down» pour allouer ses ressources de santé publique. Cependant, le pays compte 175 districts de santé dont les fortes disparités de nature épidémiologique, économique, géographique et culturelle, devraient être prises en compte pour réduire les iniquités en matière de sante publique. Comment comprendre alors que dans ces conditions, le budget des administrations publiques alloué aux différents districts de santé au Cameroun soit quasi identique alors que les défis sont si différents? L'inefficience allocative (mauvaise attribution du budget) générée par un tel système conduit vraisemblablement à une inefficience technique (mauvaise utilisation du budget). Pour la marche vers l'atteinte des objectifs du millénaire en matière de sante, il est impératif et urgent que les décideurs du secteur de la santé allouent de manière intelligente les ressources dans le système de santé.

## Opinion

Le Cameroun utilise un système de *budgétisation historique* pour allouer ses ressources. Dans ce processus, le budget de l'année A (année en cours) est calqué sur les dotations de l'année A-1 (année précédente), généralement avec quelques légères variations. Ce système assure le financement de la capacité existante (budget de moyen), mais n'assure pas nécessairement le financement des services les plus appropriés. Il ne s'assure pas non plus que les groupes prioritaires de la population bénéficient des services ni que les fonds soient utilisés de manière efficace. En outre, le système de budgétisation historique ne fournit aucune incitation quant au rendement ou aux résultats [1]. En plus de ce système de budgétisation historique, s'ajoute une approche top-down o[ugrave] c'est le niveau central qui établit un cadrage budgétaire global et fixe les enveloppes disponibles pour chaque structure et service décentralisé.

L'inefficience allocative (mauvaise attribution du budget) générée par un tel système conduit vraisemblablement à une inefficience technique (mauvaise utilisation du budget).

Le Cameroun compte 175 districts de santé présentant de fortes variabilités qui trouvent leurs origines dans la stratification sociale et dans des disparités de nature épidémiologique, économique, géographique et culturelle, pour la plus part extérieures au système de santé et qui conduisent à des iniquité. Même dans les pays à revenu élevé disposant de services de soins et de services sociaux solides et universels, des disparités sanitaires subsistent parmi les divers groupes de population [2].

Du fait de tous ces facteurs et de toutes ces réalités, comment comprendre que le budget des administrations publiques alloué aux différents districts de santé au Cameroun soit quasi identique alors que les défis sont différents?

On sait par exemple que le financement des soins destinés aux populations dispersées soulève des difficultés particulières qui sont souvent à l'origine de dépenses par habitant plus élevées que dans le cas de populations plus regroupées. Le coût unitaire par habitant est d'autant plus grand que la population couverte est faible et le coût unitaire par activité supervisée est d'autant plus grand que l'activité est faible. Il est plausible que les populations dispersées devront être subventionnées. Tout comme les populations économiquement défavorisées le devront également. Un moyen d'accélérer l'extension de la couverture des soins consisterait de ce fait à modifier les modalités d'allocation budgétaire de manière à ce qu'elles correspondent aux efforts supplémentaires nécessaires pour établir le contact avec les populations difficiles à atteindre [3]. Une approche plus rationnelle dans l'allocation des ressources permet ainsi d'améliorer l'efficience des dépenses.

Certains pays en vue d'une meilleure allocation, ont commencé à procéder à des allocations en fonction des besoins plutôt qu'en fonction de la dotation antérieure. Une telle approche fondée sur les besoins nécessite l’élaboration par le pays de formules novatrices d'allocation des ressources qui pourra se reposer sur des indicateurs démographiques tels que la taille de la population, l’âge et le genre, des indicateurs épidémiologiques tels que la charge morbide, ainsi que les degrés de pauvreté absolue ou relative.

La Tanzanie a par exemple opté depuis Janvier 2004, pour l'affectation des ressources aux districts suivant une approche qui substitue à la charge de mortalité et au niveau de pauvreté la taille de la population et la mortalité des moins de cinq ans, tout en tenant compte de la différence de coût due au fait que ces prestations de services ont lieu en milieu rural ou dans des zones faiblement peuplées. De même en Ouganda, les allocations de ressources aux districts dans le cadre du budget pour les soins de santé primaire prennent en compte l'indice de développement humain et le niveau de financement sanitaire extérieur des districts en plus de la taille de la population. Les districts qui ont des problèmes de sécurité ou qui ne disposent pas d'un hôpital bénéficient d'allocations supplémentaires [4].

Le cas du Chili est particulièrement remarquable. Les budgets sont alloués en fonction de la taille de la population mais, dans le cadre de la réforme des soins de santé primaire, ils ont été corrigés en utilisant un indice de développement humain établi par les municipalités ainsi qu'un facteur destiné à tenir compte de l'isolement des zones mal desservies [3].

Le processus de planification au Cameroun est en cours de révision pour aller vers une *planification ascendante intégrée* qui est censée s’élaborer effectivement à partir des structures de première référence et intégrer, au fur et à mesure de sa remontée les besoins spécifiques du district. Il est dès lors prévu que l'affectation des ressources soit basée sur trois critères: la taille de population, le niveau de pauvreté et l'accessibilité du district. Cependant, beaucoup de doutes persistent quant à un changement à court ou à moyen terme car, les réformes dans le pays démarrent très souvent en pompe avec effet d′annonce pour ensuite s′enliser et tomber dans l′oubli [5].

Il est donc important d'améliorer l'efficience dans l'allocation des ressources du système de santé au Cameroun en procédant à des ajustements organisationnels et en affinant les incitations économiques. Dès lors, si la question de l'influence de l'allocation du financement sur le fonctionnement du système de santé appelle naturellement à un débat sur l'avenir de ce secteur, elle interpelle de ce fait et de manière légitime les épineux problèmes de son organisation globale et de ses orientations.
